# A national policy for malaria elimination in Swaziland: a first for sub-Saharan Africa

**DOI:** 10.1186/1475-2875-10-313

**Published:** 2011-10-21

**Authors:** Simon Kunene, Allison A Phillips, Roly D Gosling, Deepika Kandula, Joseph M Novotny

**Affiliations:** 1 National Malaria Control Programme, Manzini, Swaziland, Africa; 2 Global Health Group, University of California, San Francisco, UCSF Global Health Sciences. 50 Beale Street, Suite 1200, San Francisco, CA 94105, USA; 3 Clinton Health Access Initiative and Global Health Group, University of California, San Francisco, Harare, Zimbabwe, Africa; 4 Clinton Health Access Initiative and Global Health Group, University of California, San Francisco, Mbabane, Swaziland, Africa

## Abstract

Swaziland is working to be the first country in mainland sub-Saharan Africa to eliminate malaria. The highest level of Swaziland's government recently approved a national elimination policy, which endorses Swaziland's robust national elimination strategic plan. This commentary outlines Swaziland's progress towards elimination as well as the challenges that remain, primarily around securing long-term financial resources and managing imported cases from neighbouring countries.

## Background

In March 2011, Swaziland became the first country in sub-Saharan Africa to approve a national malaria elimination policy. The technical and operational feasibility of eliminating malaria in mainland sub-Saharan Africa has been questioned [1], however, with recent declines in malaria transmission across the continent and especially in southern Africa, calls for progressive elimination have been made [2].

**Figure 1:**
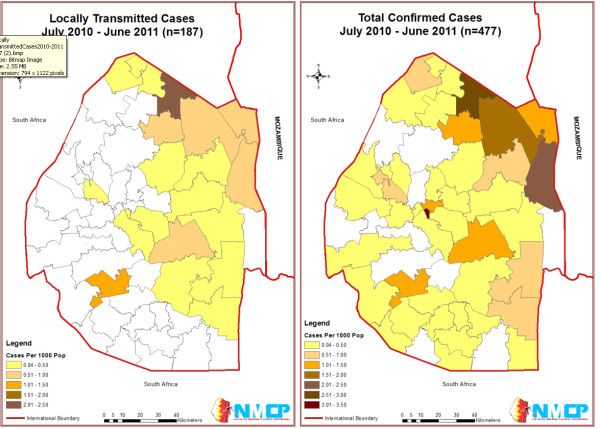
**Incidence in Swaziland per 1000 population by Inkhundla (District), 2010-2011**. ^1 ^Out of 478 confirmed cases, 376 were investigated. 187 were determined to be locally transmitted, 172 imported, and 8 were undetermined. ^2 ^Location data was available on 477 out of 478 cases.

Swaziland is a small landlocked country in southern Africa, bordering South Africa and Mozambique (Figure 1). It has one of the world's highest HIV and TB burdens and has limited national resources for health. Yet, its progressive decline in malaria and the strength of its malaria programme warrant Swaziland as the front-runner in the race to be the first mainland sub-Saharan African country to achieve elimination.

**Figure 2:**
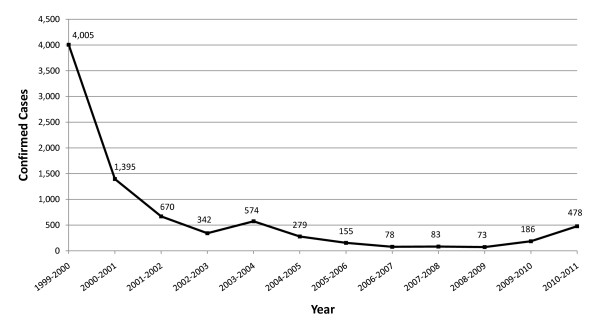
**Parasitologically Confirmed Cases in Swaziland, 1999-2011**. Prior to February 2010, all confirmed cases were diagnosed by microscopy. Parasitologically confirmed cases in 2009-10 and 2010-11 consist of cases that were confirmed by RDT and/or microscopy. Case increase in 2009-10 and 2010-2011 is due to increase in volume of patients tested for malaria.

Like other countries in the area, due to elevation and climate, the majority of Swaziland has historically had low transmission. However, in the *lowveld *ecological zone, transmission has been persistent and at times high, recording 114 cases per 1,000 population at risk in 1996 [3]. Swaziland greatly reduced the national burden of malaria; between 1999 and 2009 laboratory confirmed cases declined from 3.9 to 0.07 cases per 1000 population, as shown in Figure 2[4]. This decrease has been attributed to a scale up of vector control activities in Swaziland's at-risk region and bordering areas associated with the cross-border collaboration with Mozambique and South Africa - the Lubombo Spatial Development Initiative (LSDI) [5]. The LSDI was launched in 1999 with the goal to improve economic development in the border areas in all three countries. As malaria was viewed as an impediment to economic development, malaria control was deemed a core component of the regional partnership. The LSDI's primary malaria intervention is indoor residual spraying (IRS), specifically in high transmission areas in southern Mozambique.

Cross-border initiatives like LSDI represent a contemporary elimination strategy intended to reduce a country's importation risk and in the case of LSDI, it has been shown to lead to success towards elimination in both South Africa and Swaziland [5]. Presently, due to a lack of secure long-term funding, the continuation of LSDI remains uncertain and its potential end could threaten the progress made in all three participating countries.

With malaria control achieved through national and cross-border efforts, Swaziland has exceeded Roll Back Malaria's Abuja targets [6] and the Millennium Development Goal on malaria [7]. Recognizing Swaziland's success, the Southern African Development Community (SADC) and the African Union earmarked Swaziland for elimination by 2015 [2,8]. With the financial support of the Global Fund to Fight Aids, Tuberculosis and Malaria, technical support from the World Health Organization [9], support from SADC [10] and the Southern Africa Malaria Elimination Support Team [11], the National Strategic Plan for Elimination of Malaria in Swaziland was born.

Swaziland's strategic plan for elimination includes a robust surveillance program that identifies local and imported cases and tests all people living within a one kilometer radius of a confirmed malaria case. The Strategic Plan led to the revision of the diagnosis and treatment guidelines tailored for a low-transmission setting, scale-up of vector control interventions including distribution of long-lasting insecticide-treated nets to cover the entire malaria at-risk population, and implementation of a comprehensive health education campaign aimed to improve personal protection and treatment-seeking behaviour [4]. Since the implementation of the strategic plan in 2009, Swaziland's reported malaria incidence has decreased by 76% [12]. The reduction is mostly due to increased malaria testing, correct classification of febrile illness, and adherence to malaria test results, an important lesson for all malaria endemic countries.

Beyond a national elimination strategy, the adoption of a national malaria elimination policy is a significant step forward and a confirmation of Swaziland's commitment to the goal of being malaria free. The policy establishes clear procedures, roles and systems for all malaria stakeholders within Swaziland to contribute to the central elimination goal and ensures that the highest levels of Government remain dedicated to the elimination agenda. Support for implementation of the policy is provided by the Swaziland Malaria Elimination Advisory Group, an independent council of national malaria advisors and partners that represent 29 different constituencies and meet on a regular basis to evaluate the effectiveness of the malaria policy, monitor progress towards elimination, and revise the policy and/or strategy as appropriate. The government's commitment to elimination and preventing reintroduction fosters the necessary environment and political will for continued progress towards Swaziland's goal of becoming malaria-free by 2015.

Swaziland's substantial progress towards elimination is significant. Swaziland currently has the national and political will, operational and technical capacity, and is rapidly strengthening the systems and procedures necessary to achieve elimination. However, with the persistent risk of importation from nearby endemic countries, long-term resources for preventing reintroduction will need to be secured. Donor-funded malaria programmes, such as Swaziland's, that are progressively reducing malaria may potentially be victims of their own success. The threat of donors moving resources into other high-endemic countries could leave low-endemic countries with the risk of resurgence as seen in Madagascar [13] and Zanzibar [14]. Additional guidance to low-endemic countries on securing sustainable financing for elimination will be critical to Swaziland and other malaria-eliminating countries. With a strong collaborative effort, Swaziland is well poised to set a leading example for the rest of the sub-Saharan African region.

## Competing interests

SK is the programme manager for Swaziland's National Malaria Control Programme. DK and JMK work at the Clinton Health Access Initiative, which is in part funded by the UCSF Global Health Group. AAP and RG work at the UCSF Global Health Group. The Global Health Group exists in part to support countries that are on an evidence-based pathway towards elimination. SK, RG and AAP are members of the Malaria Elimination Group. The views and conclusions in this comment are those of the authors and do not necessarily represent the views of their employing organizations nor of the sources of funding.

## Authors' contributions

All authors contributed by guiding and shaping the messages and ideas contained in this commentary. SK shaped the key messages. The text was drafted by JMN, AAP, RG and DK, with contributions and guidance from SK. All authors took part in the review, preparation and final approval of the commentary.
